# An Assessment of Renewable Energies in a Seawater Desalination Plant with Reverse Osmosis Membranes

**DOI:** 10.3390/membranes11110883

**Published:** 2021-11-17

**Authors:** Federico Leon, Alejandro Ramos

**Affiliations:** Departamento de Ingeniería de Procesos, Universidad de las Palmas de Gran Canaria, 35417 Las Palmas de Gran Canaria, Spain; alejandro.ramos@ulpgc.es

**Keywords:** desalination, renewable energies, reverse osmosis membranes, seawater

## Abstract

The purpose of our study was to reduce the carbon footprint of seawater desalination plants that use reverse osmosis membranes by introducing on-site renewable energy sources. By using new-generation membranes with a low energy consumption and considering wind and photovoltaic energy sources, it is possible to greatly reduce the carbon footprint of reverse osmosis plants. The objective of this study was to add a renewable energy supply to a desalination plant that uses reverse osmosis technology. During the development of this research study, photovoltaic energy was discarded as a possible source of renewable energy due to the wind conditions in the area in which the reverse osmosis plant was located; hence, the installation of a wind turbine was considered to be the best option. As it was a large-capacity reverse osmosis plant, we decided to divide the entire desalination process into several stages for explanation purposes. The desalination process of the facility consists of several phases: First, the seawater capture process was performed by the intake tower. This water was then transported and stored, before going through a physical and chemical pre-treatment process, whereby the highest possible percentage of impurities and organic material was eliminated in order to prevent the plugging of the reverse osmosis modules. After carrying out the appraisals and calculating the amount of energy that the plant consumed, we determined that 15% of the plant’s energy supply should be renewable, corresponding to 1194 MWh/year. As there was already a wind power installation in the area, we decided to use one of the wind turbines that had already been installed—specifically, an Ecotecnia turbine (20–150) that produced an energy of 1920 MWh /year. This meant that only a single wind turbine was required for this project.

## 1. Introduction

Although 70% of the Earth’s surface is covered with water, only 2.5% of this is fresh water, of which 90% is inaccessible—it is found in the subsoil, in the atmosphere, and frozen in the polar caps or in glaciers. In short, less than 1% of the world’s water is suitable for consumption. Furthermore, much of this drinking water is far from populated areas, which makes its use practically impossible [1,2,3,4]. Water is an absolutely necessary resource, and, in the future, it is likely to become one of our greatest environmental problems. Therefore, desalination and water treatment facilities are becoming essential assets for economic and cultural development [5,6,7,8]. This resource has been brought to a crisis point by global warming; climate change; drought; increased birth rates (according to the latest United Nations report [3,4,9]); the advance of agricultural, livestock, and urban borders; deforestation; pollution; and mismanagement. To face this problem, engineers have designed technological alternatives that allow for the use of brackish waters as a water supply, in addition to equitable means of distributing water resources, on which we depend as a population [5,6]. By taking these technologies into account, the seas and oceans can become infinite sources of water that are suitable for consumption.

The Canary Islands are in an ideal position for the development of desalination technologies, as their geographical location and orography allow for the integration of renewable energy sources to increase the sustainability of the processes and generate a lower environmental impact on the archipelago’s ecosystem. In response to the COVID-19 crisis, the archipelago has seen its water consumption reduced, mainly due to the lack of tourists on the islands. In addition, the Government of Spain may adopt measures that could lead to water supply problems in the eastern islands due to a shortage of crude oil [9,10]. Therefore, determining ways to provide renewable energy to desalination plants is not only an environmental measure but can also be considered a measure for ensuring the survival of the islands in periods of greater vulnerability. Furthermore, when the COVID-19 crisis period ends, it is to be assumed that the flow of tourists will return to pre-pandemic levels, meaning that the demand for water will increase again.

The general objective of this study was to take advantage of the wind conditions in the area (where the average wind speed was 16.3 km/h) to generate wind energy and supply 15% of the total energy consumed by the water desalination plant located in the municipality of Granadilla de Abona, Tenerife, which employed reverse osmosis technology. To carry out this study, it was necessary to understand the processes of the desalination plant, including reverse osmosis, since this was the process by which drinking water was obtained. Osmosis is a natural process by which a solvent passes through a semipermeable membrane, moving from a diluted solution to a more concentrated one. On the other hand, reverse osmosis (RO) is a process in which the flow rate is already reduced. A force greater than the osmotic pressure is exerted through the semipermeable membrane in the opposite direction to the osmosis process. Using this method, it is possible to separate the water on one side of the membrane (concentrate) from a dilute solution low in dissolved solids on the other side of the membrane (permeate) [11,12]. The membrane is a key element in reverse osmosis, as it is the device used to treat and obtain drinking water. This process is based on forcing the passage of water and trapping impurities [12,13,14,15]. The objective of this study was to add renewable energy to a desalination plant that used reverse osmosis technology, motivated by the fact that the carbon footprint of the Canaries, where the plant was located, is much higher than that of mainland Spain. This is due to the use of non-renewable energies, mainly fuel, gasoil, and gas. Solar and wind power have great potential, as innovative renewable energy sources, to lead to a reduction in the use of greenhouse gases. 

## 2. Materials and Methods

One of the most important aspects to consider is the selection of the correct membrane according to the process that will be carried out. In addition, it is important to take into account factors related to the installation and its cost [16,17].

In this section, we describe the design equations for micro, ultra, and nanofiltration membranes used for pre-treatment, as well as the equations that define reverse osmosis itself.

There are several different types of membrane technology. The most commonly exploited are microfiltration (MF), ultrafiltration (UF), and nanofiltration (NF). These types of membrane create new possibilities when it comes to the pre-treatment of seawater [17,18,19,20]. The flow of water (J) through the MF and UF membranes is described by the following equation:

(1) $$J=\frac{\Delta P}{\eta *R_{T}}$$

where:

- J→ water flow $\left(m^{3}/m^{2}\cdot s\right)$;
- ΔP→ differential pressure or applied transmembrane pressure (PTM) in $N/m^{2}$;
- η→ dynamic viscosity $N\cdot s/m^{2}$;
- $R_{T}\to$ total membrane resistance (1/m).

The net applied pressure (PNT) is proportional to the flow of water and inversely proportional to the viscosity, temperature, and total resistance of the membrane [21,22,23,24]. The non-crossflow rate at transmembrane pressure can be determined from the following equation:

(2) $$PTM=P_{i}-P_{p}$$

where:

- $P_{i}\to$ inlet pressure;
- $P_{p}\to$ permeate pressure.

In cases where the flow is crossed, the following equation is used [21,22,23,24]:

(3) $$PMT=\frac{P_{e}*P_{s}}{2}-P_{p}$$

where:

- $P_{e}\to$ diaphragm inlet pressure;
- $P_{s}\to$ diaphragm outlet pressure.

Finally, considering the previous equations, the flow as it corresponds to the membrane can be determined by the following equation [25,26,27,28,29]:

(4) $$Q_{P}=J*S$$

where:

- S→ available membrane surface $(m^{2});$
- $Q_{P}\to$ treated or permeated water flow (L/h).

**Figure 1:**
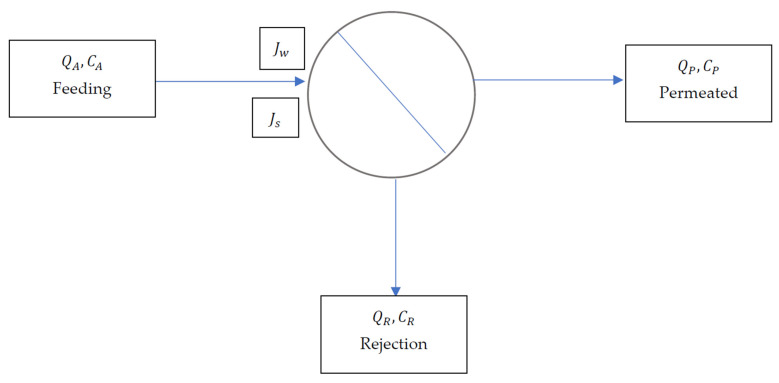
Simplification of the desalination process [30].

Reverse osmosis formulas using a solution–diffusion model:

The formulas used for the calculations of the reverse osmosis process are based on the solution–diffusion model. In this model, there is a salt flow step (*J_s_*) and a water flow step (*J_w_*), as shown in Figure 1.

One of the most important equations used in this study is the water or solvent flow equation, which is as follows [30]:

(5) $$J_{w}=A*\left(\Delta P-\Delta \pi \right)$$

where:

- $J_{w}\to$ water flow in liters $\left(m^{2}/h\right)$;
- A→ membrane permeability coefficient $\left(L/m^{2}/\mathrm{bar}\right)$;
- ΔP→ transmembrane differential pressure, bar;
- Δπ→ osmotic pressure difference, bar.

To correctly carry out these calculations, it is necessary to consider the concept of net working pressure (NDP).

From this formula, it follows that the higher the net working pressure, the higher the productivity of the membrane.

Considering these points, we present the following equation [30]:

(6) $$J_{s}=B*\Delta C$$

where:

- $J_{s}\to$ salt flow ($\mathrm{kg}/m^{2}/s$);
- B→ mass transfer coefficient (m/s);
- ΔC→ transmembrane differential mean concentration $\left(\mathrm{kg}/m^{3}\right)$.

The characteristics of the membranes depend on factors A and B, in addition to temperature, pH, conversion factors, and salinization concentrations, which can be modified.

For the development of a reverse osmosis plant, therefore, it should be kept in mind that the higher the concentration of salts in the feed is, the greater the passage of the salts will be, which will cause an increase in the salinity of the permeate [31,32,33,34,35,36,37].

Material balance.

Based on Figure 1, two types of balance can be described [31,32,33,34,35,36,37]:

1. Solvent balance:
  (7) $$Q_{A}=Q_{p}+Q_{r}$$
2. Solute balance:
  (8) $$Q_{A}*C_{A}=Q_{p}*C_{p}+Q_{r}*C_{r}$$

where:

- $Q_{A}\to$ feed flow $\left(m^{3}\cdot h^{-1}\right)$;
- $C_{A}\to$ feed solute concentration $\left(\mathrm{kg}\cdot m^{-3}\right)$;
- $Q_{p}\to$ permeate flow $\left(m^{3}\cdot h^{-1}\right)$;
- $C_{p}\to$ permeate solute concentration $\left(\mathrm{kg}\cdot m^{-3}\right)$;
- $Q_{r}\to$ reject or permeate flow $\left(m^{3}\cdot h^{-1}\right)$;
- $C_{r}\to$ solute concentration in the reject or concentrate $\left(\mathrm{kg}\cdot m^{-3}\right)$.

Conversion factor and concentration factor.

The percentage of permeation is obtained from a certain feed flow and can be expressed as the ratio of the permeate flow to the contribution flow that reaches the membrane. This is also called the conversion factor [31,32,33,34,35,36,37].

(9) $$Y=\frac{Q_{p}}{Q_{A}}*100=\left(1-\frac{Q_{p}}{Q_{A}}\right)*100$$

The concentration factor is directly related to the conversion factor, as shown by the following formula [31,32,33,34,35,36,37]:

(10) $$FC=\left(\frac{1}{1-Y}\right)$$

where:

- FC→ concentration factor;
- Y→ conversion factor.

### Salt Rejection Factor (R) and Salt Passage (SP)

The formula used for determining the salt rejection percentage is as follows [31,32,33,34,35,36,37]:

(11) $$R=\frac{C_{A}-C_{p}}{C_{A}}*100$$

- R→ salt rejection percentage.

As a result, the percentage of the passage of salts can be calculated via the following equation [31,32,33,34,35,36,37]:

(12) SP(%)=100−R

## 3. Results and Discussion

This work assessed the contribution of renewable energy to a desalination plant that used reverse osmosis technology. It was found that the feed water came from the sea with a high salinity and was filtered at a low pressure in the pre-treatment system through reverse osmosis membranes, which carried out the desalination process. This produced potable water and high-concentration brine, which was returned to the sea. The brine pressure energy was recovered by energy recovery devices, and the permeate water was sent to a wastewater plant, before being returned to the sea or used for irrigation purposes. In fact, it was also possible to reuse the reverse osmosis membranes as ultrafiltration filters after controlled oxidation or to recycle them as plastic [1,2,3,6,37].

We determined the following environmental benefits (Table 1) and strategic and socioeconomic advantages (Table 2 and Table 3) of renewable energy compared with conventional energy:

In the Canary Islands, a mixture of renewable and conventional energy is consumed. The electrical power installed in the Canary Islands amounted to 3308.6 MW, which was divided as shown in Table 4 [3,37,38]:

The two energy sources on which this project focused, photovoltaic and wind energy, were determined by the figures shown above for the energy supplies currently used in the archipelago [3,37,39].

Table 5 shows the advantages and disadvantages of photovoltaic solar energy installations.

The following considerations were made concerning the results obtained for the design and creation of a photovoltaic installation:

*Intensity of the module*

Data from the stress curve of the solar module were calculated via the following equations [39]:

(13) $$FF=\frac{I_{mpp}*V_{mpp}}{I_{sc}*V_{oc}}$$

(14) $$P_{mpp}=FF*I_{sc}*V_{oc}$$

where:

- $V_{mpp}\to$ voltage at the maximum power point;
- $I_{mpp}\to$ intensity produced at the maximum power point;
- $V_{oc}\to$ unloaded voltage;
- $I_{sc}\to$ short-circuit intensity;
- $P_{mpp}\to$ maximum produced power under standard conditions (STC);
- FF→ form factor.

*Peak nominal potential*

The power of the photovoltaic installation was determined by the peak rated power, which was conditioned by:

- Irradiation $\to \;G_{stc}=1000\;W/m^{2}$ at normal incidence;
- Temperature in normal conditions;
- Air mass (AM), which usually has a value of 1.5, but can be modified by atmospheric pressure (P) or the zenith angle (h) according to the following equation:
  (15) $$AM=\frac{P}{\left(P_{o}*sen\left(h\right)\right)}$$

Table 6 shows the definitions of the parts of a wind turbine. Moreover, as with photovoltaic solar energy, wind energy has advantages and disadvantages, and these are shown in Table 7 [39].

**Figure 2:**
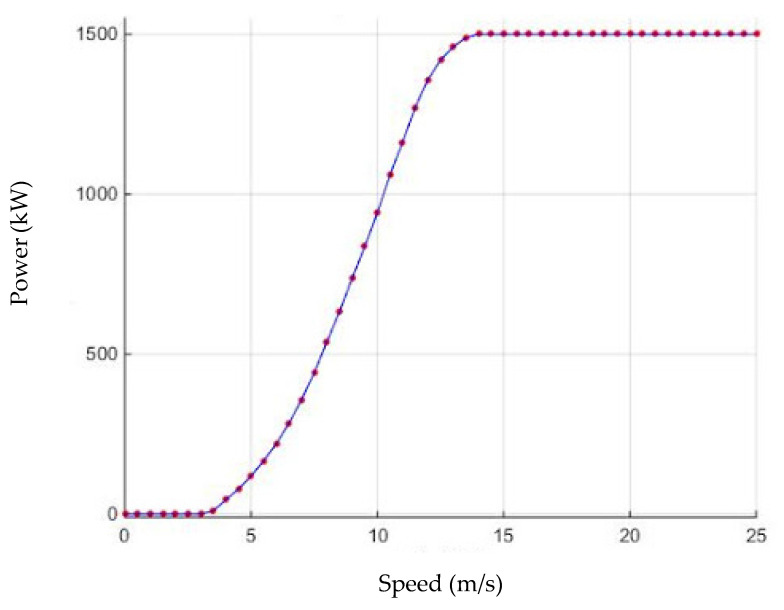
Energy produced by a wind turbine.

For a wind turbine to operate, a minimum wind speed is required. Because the wind speed changes depending on the height, it was necessary to consider Hellmann’s exponential law [39]:

(16) $$V\left(h\right)=V_{o}*{\left(\frac{h}{h_{o}}\right)}^{\alpha }$$

where:

- V(h)→ wind speed at the chosen height;
- $V_{o}\to$ known wind speed at the reference height;
- h→ chosen height;
- $h_{o}\to$ reference height;
- α→ site-dependent and determined by the following Table 8:

Three types of wind speed were considered to understand the operation of a wind turbine (Figure 2):

- Connection speed: the speed at which energy is generated.
- Nominal speed: the optimal speed at which the wind turbine reaches its rated power.
- Disconnect speed: the high speed at which the wind turbine disconnects and stops generating power.

*Wind power*

The power generated by the wind turbine depends on the wind speed in the area of installation, taking into account the height, the sweeping section of the blades, the density of the air, and other variables. The equation used for determining the power generated by a wind turbine is as follows [39]:

(17) $$P=\frac{1}{2}*\rho *A*V^{3}$$

where:

- ρ→ wind density = 1225 $\mathrm{kg}\cdot m^{-3}$;
- P→ power (W);
- A→ swept area:
  (18) $$A=\pi *r^{2};$$
- V→ Wind speed $\left(m\cdot s^{-1}\right)$.

*Turbine power: power coefficient* $C_{p}$

The mechanical rotational energy is also called the power coefficient, which is determined by the following equation [39]:

(19) $$P_{t}=C_{p}*P$$

*Betz’s Limit*

Betz’s limit, or law, prevents all available power from being extracted from the wind as it passes through a wind turbine’s rotor. The wind that passes through the wind turbine is slowed down, thus it leaves at a lower speed than that at which it entered. 

**Figure 3:**
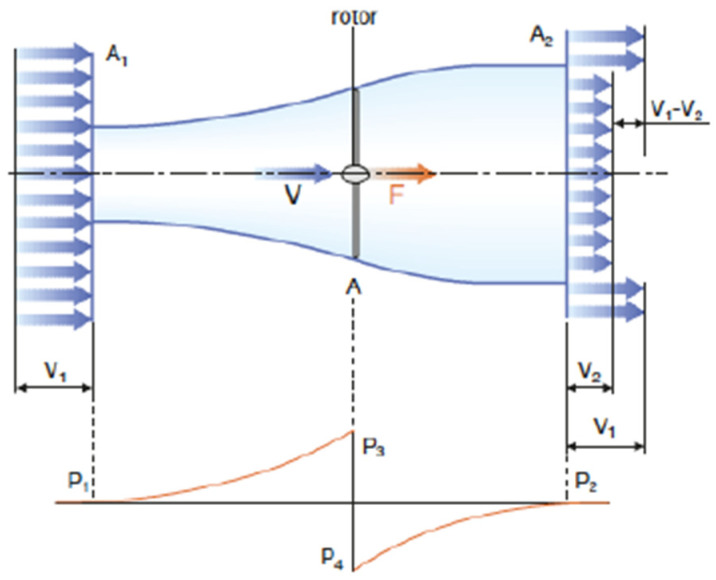
Demonstration of Betz’s theorem.

For the turbine shown in Figure 3, the performance peaks at $\frac{v_{2}}{v_{1}}=\frac{1}{3}$ and the maximum power value is 0.59—i.e., the rotor power will never exceed 59%.

*Estimating energy productivity*

The productivity of the wind turbine was determined by the following equation:

(20) $$E=8760*\int_{0}^{\infty }P\left(v\right)*f\left(v\right)*dv$$

where:

- 8760→ number of hours corresponding to one year;
- P(v)→ power (kW);
- f(v)→ Weibull distribution, which was used to estimate the energy production of the wind turbine. To obtain this figure it was necessary to have a series of data pertaining to the wind speeds in the area where the wind turbine was to be installed.

*Renewable energy results*

Table 9 itemizes several important features of the reverse osmosis process. 

Further integration of renewable energy sources is recommended in order to achieve greater sustainability and reduce the environmental impact of the desalination process. Open water intakes are used for large-capacity plants and these were also considered for this installation.

In this section, we calculated the amount of energy consumed by the plant and the amount of renewable energy that should be supplied.

To do this, we considered the number of elements involved in the plant’s processes and the nominal power consumed by each element, which was obtained from their data sheets. We estimated an average of 12 h of daily work, since reverse osmosis membranes take approximately 3 to 4 h to fill.

The following Table 10 lists the data for each element:

The hours of work per day and the energy consumed are specified in Table 11.

**Figure 4:**
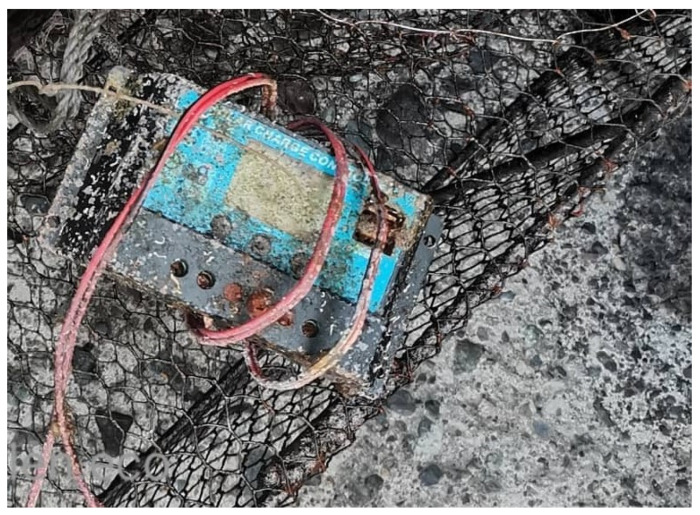
Solar power charging connector recovered via underwater retrieval in Los Abrigos, Tenerife.

As specified in previous sections, we proposed that renewable energy should make up 15% of the plant’s energy supply, as shown in Table 12:

The Granadilla de Abona industrial city in Tenerife is an area known for the presence of strong and constant winds. Because of this, we decided to discard solar energy as a possible solution, since fragments of photovoltaic installations were found in the area after dredging (Figure 4).

The proposed wind energy system will be interconnected with the plant’s main energy supply from the electricity grid, meaning that 15% of the total energy supply will be generated by the wind turbine. This figure of 15% may vary, as the optimal performance of the wind turbine cannot be guaranteed.

This interconnectivity would bring with it several advantages, such as rendering energy storage elements unnecessary.

These processes are covered by Royal Decree 1955/2000 and Royal Decree 1699/2011, the latter of which allows for the sale of energy in the case of an energy surplus.

The energy required by the plant is 1194 megawatt hours per year. The ITER provides a range of wind turbines, Table 3, the Ecotecnia wind turbine was selected for this project based on the consideration of the characteristics of the location where the installation will be developed in Table 13 (20–150):

It was decided that a single wind turbine will be used for this project, since it will provide the energy necessary to supplement the running of the plant.

## 4. Conclusions

The object of this study was to add a renewable energy supply to a desalination plant that uses reverse osmosis technology. The development of renewable energy sources was recommended in order to achieve greater sustainability and reduce the environmental impact of the desalination process. We considered the introduction of wind turbines to provide the energy needed for the correct development of the installation. Regarding the LCIA (life cycle impact assessment), it was found that the feed water came from the sea with a high salinity and it was filtered at a low pressure in the pre-treatment system through the reverse osmosis membranes, which carried out the desalination process.

It was determined that the Granadilla desalination plant produced potable water and high-concentration brine, which was returned to the sea. The brine pressure energy was recovered by energy recovery devices and the permeate water was sent to a wastewater plant, before being returned to the sea or used for irrigation purposes. It was also possible to reuse the reverse osmosis membranes as ultrafiltration filters after controlled oxidation or to recycle them as plastic. For this large-capacity reverse osmosis plant, it was recommended that the whole desalination process should be divided into several stages.

The Canary Islands are in an important position for the development of desalination technologies; therefore, the development of renewable energy sources was recommended in order to achieve greater sustainability and reduce the environmental impact of the desalination process. We calculated the amount of energy consumed by the SWRO plant and the amount of renewable energy that could be supplied. For this project in the Canary Islands, we considered the introduction of wind turbines to provide the energy needed for the running of the plant.

## Figures and Tables

**Table 1:** Environmental benefits.

Environmental	
Renewable	Conventional
Does not produce emissions of polluting gases	Obtained from fossil fuels
Does not generate hard-to-treat waste	Generates waste that is difficult to remove or that poses a threat to the environment
	Is finite

**Table 2:** Strategic advantages.

Strategic	
Renewable	Conventional
Is indigenous	Exists in a limited number of countries
Does not depend on other countries for production	Requires energy imports

**Table 3:** Socioeconomic advantages.

Socioeconomic	
Renewable	Conventional
Creates jobs	Does not create as many jobs as renewables
Allows for countries to develop their own technologies	Mostly requires imported technology
Contributes significantly to the interterritorial balance by centering production in rural areas	Located in developed areas

**Table 4:** Electrical power installed in the Canary Islands.

Electrical Power Installed in the Canary Islands (MW)		
Thermal origin	Thermal power plants	2606.4
	Refinery	25.9
	Cogeneration	64.1
	Total	2696.4
Renewable origin	Wind	397.3
	Photovoltaic	186.5
	Mini hydraulics	2.0
	Hydroeolic	22.8
	Biogas	3.7
	Total	612.3
Overall total		3308.7

**Table 5:** Advantages and disadvantages of photovoltaic solar energy.

Advantages	Disadvantages
Long service life	High initial cost
Reduced costs with maintenance	Power generation is not constant due to the variability of the power source
Modularity	

**Table 6:** Definitions of the parts of a wind turbine.

Parts of the Wind Turbine	
Blade	Like the wings of an airplane, built with relatively light materials
Nacelle	Contains the different components of the wind turbine. Its exterior has a vane and an anemometer that control the turbine
	
Rotor axle and Controller	Join together the blades and the bushing. Transform kinetic energy into mechanical energy
Tower	Supports the nacelle and the rotor
Generator	Transforms mechanical energy into electrical energy
Guidance system	Places the rotor perpendicular to the wind
Foundation	High-strength platform that provides support for the wind turbine
Electrical regulation system	Maintains rotation speed and limits wind power

**Table 7:** Advantages and disadvantages of wind energy.

Advantages	Disadvantages
Low cost of generation	Wind is not guaranteed
Creates skilled jobs	Energy cannot be stored
Energy independence	Impacts on the landscape
Increases wealth in rural areas	Affects birds

**Table 8:** Values of α.

Type of Land	α
Smooth (sea, sand, snow)	0.10–0.13
Moderately rough (crops)	0.13–0.20
Rough (forests, buildings)	0.20–0.27
Very rough (cities)	0.27–0.40

**Table 9:** Features of reverse osmosis installation.

Reverse Osmosis Plant Characteristics	
Plant Production ($m^{3}/\mathrm{day}$)	10,000
Stages of process	5
Operating time (h)	24
Water origin	Seawater
Intended product	Drinking water
Salt concentration (mg/L)	36,662,391
Design temperature (°C)	20
Minimum temperature (°C)	18
Maximum temperature (°C)	23

**Table 10:** Total power consumed by each element.

Element	Total Power (kW) $\left(\mathrm{Unit}*P_{N}\right)$
Sand filter	325
Membrane	11,115
Energy recovery	96
Catchment pump	550
Transfer pump	550
High-pressure pump	350
Booster pump	150
Chemical wash pump	55
Water pump product	375
Total	1,832,115

**Table 11:** Energy consumed per day for each element.

	Hours of Work Per Day (h)	Energy Consumed Per Day (kWh) $\left(h*P_{N}\right)$
Sand filter	12	390
Membrane	10	111,150
Energy recovery	10	960
Catchment pump	12	6600
Transfer pump	12	6600
High-pressure pump	12	4200
Booster pump	12	1800
Chemical wash pump	10	550
Water pump product	16	600
	Total	21,811,150

**Table 12:** Renewable energy supply.

Total Power (kW)	Energy Consumed (kWh)	Renewable Energy Supply (kWh/día)
1,832,115	21,811,150	3,271,673

**Table 13:** Ecotecnia wind turbine data (20–150).

Wind Turbine Characteristics	
Manufacturer	Ecotecnia
Diameter (m)	20
Height (m)	30
Power (kW)	150
Rotor	Horizontal axis
Energy generated per year (MWh)	1920
Number of blades	3
Generator	Asynchronous

## Abbreviations

RO	Reverse osmosis;
MF	Microfiltration;
UF	Ultrafiltration;
NF	Nanofiltration;
J	$\mathrm{Water}\;\mathrm{flow}\;\left(m^{3}/m^{2}\cdot s\right)$;
ΔP	$\mathrm{Differential}\;\mathrm{pressure}\;\mathrm{or}\;\mathrm{applied}\;\mathrm{transmembrane}\;\mathrm{pressure}\;(\mathrm{PTM})\;\mathrm{in}\;N/m^{2}$;
η	$\mathrm{Dynamic}\;\mathrm{viscosity}\;N\cdot s/m^{2}$;
$R_{T}$	Total, membrane resistance (1/m);
$P_{i}$	Inlet pressure;
$P_{p}$	Permeate pressure;
$P_{e}$	Diaphragm inlet pressure;
$P_{s}$	Diaphragm outlet pressure;
S	$\mathrm{Available}\;\mathrm{membrane}\;\mathrm{surface}\;(m^{2})$;
$Q_{P}$	Treated or permeated water flow (L/h);
$J_{w}$	$\mathrm{Water}\;\mathrm{flow}\;\mathrm{in}\;\mathrm{litres}\;(m^{2}/h)$;
A	$\mathrm{Membrane}\;\mathrm{permeability}\;\mathrm{coefficient}\;(L/m^{2}/\mathrm{bar})$;
ΔP	Transmembrane differential pressure, bar;
Δπ	Osmotic pressure difference, bar;
$J_{s}$	$\mathrm{Salt}\;\mathrm{flow}\;(\mathrm{kg}/m^{2}/s)$;
B	Mass transfer coefficient (m/s);
ΔC	$\mathrm{Transmembrane}\;\mathrm{differential}\;\mathrm{mean}\;\mathrm{concentration}\;(\mathrm{kg}/m^{3})$;
$Q_{A}$	$\mathrm{Feed}\;\mathrm{low}\;(m^{3}\cdot h^{-1})$;
$C_{A}$	$\mathrm{Feed}\;\mathrm{solute}\;\mathrm{concentration}\;\left(\mathrm{kg}\cdot m^{-3}\right)$;
$Q_{p}$	$\mathrm{Permeate}\;\mathrm{flow}\;(m^{3}\cdot h^{-1})$;
$C_{p}$	$\mathrm{Permeate}\;\mathrm{solute}\;\mathrm{concentration}\;\left(\mathrm{kg}\cdot m^{-3}\right)$;
$Q_{r}$	$\mathrm{Reject}\;\mathrm{or}\;\mathrm{permeate}\;\mathrm{flow}\;(m^{3}\cdot h^{-1})$;
$C_{r}$	$\mathrm{Solute}\;\mathrm{concentration}\;\mathrm{in}\;\mathrm{the}\;\mathrm{reject}\;\mathrm{or}\;\mathrm{concentrate}\;\left(\mathrm{kg}\cdot m^{-3}\right)$;
FC	Concentration factor;
Y	Conversion factor;
R	Salt rejection, %;
SP	Salt passage, %.
